# High intralocus variability and interlocus recombination promote immunological diversity in a minimal major histocompatibility system

**DOI:** 10.1186/s12862-014-0273-1

**Published:** 2014-12-20

**Authors:** Anthony B Wilson, Camilla M Whittington, Angela Bahr

**Affiliations:** Institute of Evolutionary Biology and Environmental Studies, University of Zurich, Winterthurerstrasse 190, 8057 Zurich, Switzerland; Department of Biology, Brooklyn College and The Graduate Center, City University of New York, 2900 Bedford Avenue, Brooklyn, New York 11210 USA; School of Biological Sciences, University of Sydney, Heydon-Laurence Building A08, Sydney, NSW 2006 Australia; Institute of Medical Molecular Genetics, University of Zurich, Wagistrasse 12, 8952 Schlieren, Switzerland; Department of Biology, Brooklyn College, 2900 Bedford Avenue, Brooklyn, New York 11210 USA

**Keywords:** Gene conversion, Major histocompatibility complex, Natural selection, Recombination, Sexual selection, Syngnathidae

## Abstract

**Background:**

The genes of the major histocompatibility complex (MHC/MH) have attracted considerable scientific interest due to their exceptional levels of variability and important function as part of the adaptive immune system. Despite a large number of studies on MH class II diversity of both model and non-model organisms, most research has focused on patterns of genetic variability at individual loci, failing to capture the functional diversity of the biologically active dimeric molecule. Here, we take a systematic approach to the study of MH variation, analyzing patterns of genetic variation at MH class IIα and IIβ loci of the seahorse, which together form the immunologically active peptide binding cleft of the MH class II molecule.

**Results:**

The seahorse carries a minimal class II system, consisting of single copies of both MH class IIα and IIβ, which are physically linked and inherited in a Mendelian fashion. Both genes are ubiquitously expressed and detectible in the brood pouch of male seahorses throughout pregnancy. Genetic variability of the two genes is high, dominated by non-synonymous variation concentrated in their peptide-binding regions. Coding variation outside these regions is negligible, a pattern thought to be driven by intra- and interlocus recombination. Despite the tight physical linkage of MH IIα and IIβ loci, recombination has produced novel composite alleles, increasing functional diversity at sites responsible for antigen recognition.

**Conclusions:**

Antigen recognition by the adaptive immune system of the seahorse is enhanced by high variability at both MH class IIα and IIβ loci. Strong positive selection on sites involved in pathogen recognition, coupled with high levels of intra- and interlocus recombination, produce a patchwork pattern of genetic variation driven by genetic hitchhiking. Studies focusing on variation at individual MH loci may unintentionally overlook an important component of ecologically relevant variation.

## Background

The genes of the major histocompatibility complex (MHC) are an essential component of the adaptive immune system, and among the most variable loci in the vertebrate genome [1]. Classical major histocompatibility (MH) class I and II genes are responsible for the recognition of foreign antigens [2,3], and the peptide-binding regions (PBR) of these genes represent some of the most striking examples of balancing selection [4]. MH class II molecules are protein dimers produced from the combination of class IIα and IIβ genes, which together form the immunologically-active peptide-binding cleft involved in the recognition of extracellular pathogens [5]. The high variability of the peptide-binding regions of these two genes ensures that hosts can recognize a broad spectrum of pathogens, and these regions are consequently subject to strong positive selection.

Despite the fact that antigen recognition by MH class II is determined by a protein complex including both IIα and IIβ molecules, the majority of studies have focused on only a single component of this complex (typically MH IIβ), neglecting the fact that antigen discrimination is likely to be realized via unique permutations of MH IIα and IIβ alleles in the host. This gene-centric approach to the analysis of MH diversity has been premised on the assumption that allelic variability at class IIα loci is negligible relative to that found at class IIβ genes. While genetic variability of MH class IIα is comparatively low in human populations [6], this hypothesis has not been well investigated in other vertebrate taxa.

The majority of vertebrates carry multiple copies of classical MH loci [7], complicating efforts to study intra- and interlocus allelic variability in natural populations. In contrast to the tightly linked structure of major histocompatibility genes in mammals, MH genes are unlinked in teleost fishes, and members of this group show considerable variation in the number and structure of MH class I and II loci [8], providing a powerful comparative model for the study of evolutionary immunology. A recent study comparing patterns of allelic variability of MH IIα and IIβ loci in salmonid fishes with a minimal MH class II system found high levels of genetic variation at both loci, diversity that could serve to maintain the efficiency of pathogen recognition despite the absence of additional gene copies [9]. Unfortunately, given the relative paucity of studies directly investigating allelic variability of both components of the MH II molecule, it remains unclear whether the high genetic diversity found at both loci in salmonids is a lineage-specific phenomenon associated with their minimal MH system, or whether it reflects a broader pattern relevant for understanding MH class II function in other taxa.

Seahorses (*Hippocampus* spp.) are a genus of marine fishes well known for their unique form of reproduction, male pregnancy, in which males provide all parental care after fertilization in highly specialized brooding structures located on their ventral surface [10]. Recent research on the pot-bellied seahorse (*H. abdominalis*) found evidence of a single MH IIβ locus with high allelic diversity in this species [11], and a highly skewed distribution of non-synonymous variation across the gene [12]. Behavioral studies of *H. abdominalis* indicate that females can discriminate male MH IIβ genotype using olfactory cues, and use this information when making mate choice decisions [13]. Males, in contrast, show a lack of MH IIβ olfactory discrimination.

Transcriptome profiling of the seahorse indicates that this species carries a minimal MH class II system, with single expressed copies of both MH IIα and β. Intriguingly, while these genes are intact in the seahorse, pipefish (*Syngnathus typhle*) have apparently lost MH class II genes as well as CD4^+^, a critical component of MH II-associated T-helper cells [14]. Given the close relationship between seahorses and pipefish [15,16], the loss of MH II function in the pipefish appears to be a relatively recent evolutionary event, one which would be expected to have an important impact on adaptive immune function in this group.

In this study, we identify the genomic structure and pattern of inheritance of major histocompatibility class II genes in the seahorse, and use population-level screening to examine the distribution of genetic variation across their peptide-binding regions. We characterize tissue-specific patterns of expression of the two loci, with particular emphasis on the male brood pouch, the reproductive organ associated with male pregnancy in this species. Finally, MH IIα and IIβ data are used to reconstruct the functionally important peptide-binding cleft of the molecule via homology modelling to the known crystallographic structure of the mature protein. Despite the tight physical linkage between MH IIα and IIβ in the seahorse, composite allele profiles reveal high levels of functional diversity at both loci, and a strong signature of intra- and interlocus recombination, insights essential for understanding the structure, function and evolution of the biologically-active protein complex.

## Results

### The seahorse possesses a minimal MH II system

**Figure 1:**
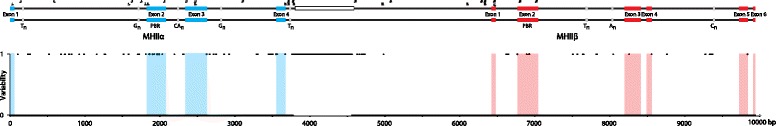
**Sequence variability map of the MH class II region of the seahorse, based on the sequencing of alleles from a single heterozygous individual.** Intron-exon structures of MH class IIα and IIβ are indicated as well as SNPs distinguishing the two full-length alleles (i.e. variability = 1). Note the presence of regions of repetitive sequence (A_n_, C_n_, CA_n_, G_n_ and T_n_) as well as a 786 bp deletion in the region between the IIα and IIβ loci. Primers used in the amplification and sequencing of this region are indicated (see Table 1 for primer sequences). Full length sequences have been deposited in GenBank (KP259908, KP259909).

Full length cDNA and gDNA amplification of MH IIα in the seahorse revealed the existence of a single four exon MH IIα locus in this species. PCR reactions designed to bridge the full MH class II gene region produced two PCR products in an individual heterozygous for MH IIβ, both of which were successfully cloned and sequenced using primers distributed across the length of the sequence (Table 1). Analysis of cloned products identified extended single bp and tandem repeats in intronic regions of both MH IIα and IIβ, along with a large 786 bp indel located in the intergenic region (Figure 1). The full length MH class II region of the seahorse is 9,130 - 9,884 bp in length, and contains a single class IIα and IIβ locus (Figure 1). Exonic variation is concentrated in the putative peptide binding regions (PBR) of the two MH loci (Figure 1), and while overall variation in coding regions is low, all exonic SNPs in the sequenced individual are non-synonymous (MH IIα Exon 2 (PBR): 11/11 SNPs non-synonymous, MH IIα Exon 4: 3/3, MH IIβ Exon 2 (PBR): 4/4, MH IIβ Exon 3: 1/1; MH IIβ Exon 5: 1/1).

**Table 1:** **Primers (5′ → 3′) used to amplify and sequence major histocompatibility class II genes in** ***H. abdominalis***

**#**	**Name**	**Sequence 5′-3′**	**Location**
1	MHIIα-E1F3	TTACTCCGTTGCGGCGACGCC	Exon 1
2	MHIIα-I1R	CGTCTGTACAATACTTCCGTAC	Intron 1
3	MHIIα-I1F	CAGTTACCAGGACAATGAC	Intron 1
4	MHIIα-I1F2	CAAAAAGCGGTGCTTATCGAG	Intron 1
5	MHIIα-I1R2	GCTTTAACTTGAGATACAAGTACC	Intron 1
6	MHIIα-I1R3	CACCAACAGTGAAAAACCACAAG	Intron 1
7	MHIIα-I1F3	CTTGCGGGGGAAACGGCGAAG	Intron 1
8	MHIIα-E2F*	GACGTCATCCACACAGACATGC	Exon 2
9	MHIIα-E2R2	GATTCTAGTGGCTGATCCTTCAATGC	Exon 2
10	MHIIα-E3R*	TGATGGGATGGGAGGGAGGATC	Exon 3
11	MHIIα-E3F	AGACCTTCCAACTGGACTTCAC	Exon 3
12	MHIIα-E3R2	GTGAAGTCCAGTTGGAAGGTCTGG	Exon 3
13	MHIIα-I3R	CACGTTATTTATCATCGGCATAG	Intron 3
14	MHIIα-E4F	CATCAAAGGAAACCAATGCAACTG	Exon 4
15	MHIIα-3UTRR	GTGAGGACATAATGTGCGCCACC	3′ UTR
16	MHIIα-3UTRF	GGTGGCGCACATTATGTCCTCAC	3′ UTR
17	MHIIβ-5UTRR5	GCAATACACTGGGGTTCTCTG	5′ UTR
18	MHIIβ-5UTRF2	CAGTGTATTGCAGAACATGG	5′ UTR
19	MHIIβ-5UTRR6	TATCGGTACTGGCCCATCC	5′ UTR
20	MHIIβ-5UTRF	GCGACCAAAATCCGTTCCAG	5′ UTR
21	MHIIβ-5UTRR4	GGCGTGTTTGTTGTGATTTACAC	5′ UTR
22	MHIIβ-5UTRF3	GCTTTGTGCAACTAATTGTGC	5′ UTR
23	MHIIβ-5UTRR2	GGATCCAGTCTAATCTGAATCCCG	5′ UTR
24	MHIIβ-5UTRR	CCAACCTGAGCACAAACTTG	5′ UTR
25	MHIIβ-UTR5F	CAAGTTTGTGCTCAGGTTGG	5′ UTR
26	MHIIβ-E1R	AAGGTGAGGAAAAGGAGGC	Exon 1
27	MHIIβ-E1F2*	GCCTCCTTTTCCTCACCTTC	Exon 1
28	MHIIβ-E2R2	GATGTCATTCTGGTCACTCGAGTT	Exon 2
29	MHIIβ-E2R*	GAGCGCACTTTCGTAGTCAA	Exon 2

*RT-PCR primers.

**Figure 2:**
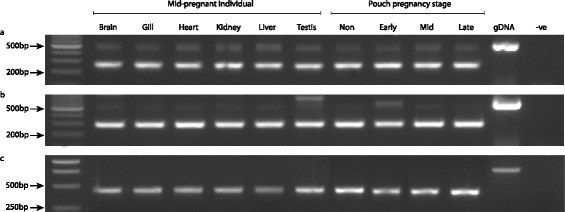
**MH class IIα and IIβ genes are ubiquitously expressed in the seahorse.** Gel images showing results of RT-PCR on a range of seahorse tissues: **(a)** MH class IIα, **(b)** MH class IIβ, **(c)** Beta-actin (positive control).

MH IIα and IIβ are constitutively expressed, and present in the brood pouch of both pregnant and non-pregnant male seahorses (Figure 2). RT-PCR analysis showed no differences in expression levels of either gene across different tissues or different stages of pregnancy.

**Figure 3:**
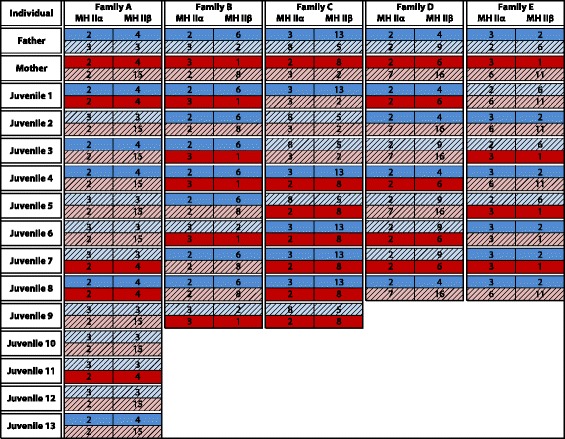
**Mendelian inheritance of MH class II alleles in five families of known parentage.** Parental alleles are color-coded for ease of discrimination in offspring (Paternal alleles: light/dark blue; Maternal alleles: light/dark red). Blockwise inheritance of parental MH alleles provides direct evidence of tight physical linkage.

Analysis of the MH class IIα and IIβ PBR in five families of known parentage demonstrates that genetic variation at both loci is inherited in a Mendelian fashion (Figure 3). Analyses of allelic phase in parents and offspring support the tight physical linkage of MH IIα and β, with no evidence of germline recombination evident in the dataset (Figure 3).

### Evidence of balancing selection on the MH IIα PBR

Direct sequencing of the putative MH IIα peptide-binding region in a population sample of 101 individuals identified a high level of functionally important genetic variation, with 18 alleles observed in the 246 bp region, including 25 substitutions at 24 polymorphic nucleotide sites. Nucleotide diversity (π) in the population sample equaled 0.030 ± 0.007, similar to the level of genetic variation found at MH IIβ of the seahorse (π = 0.031 ± 0.007) [11]. 84% of individuals were heterozygous for MH IIα (85 of 101), while 16% were homozygous, values consistent with Hardy-Weinberg expectations (HWE Exact Test: p = 0.61).

**Figure 4:**
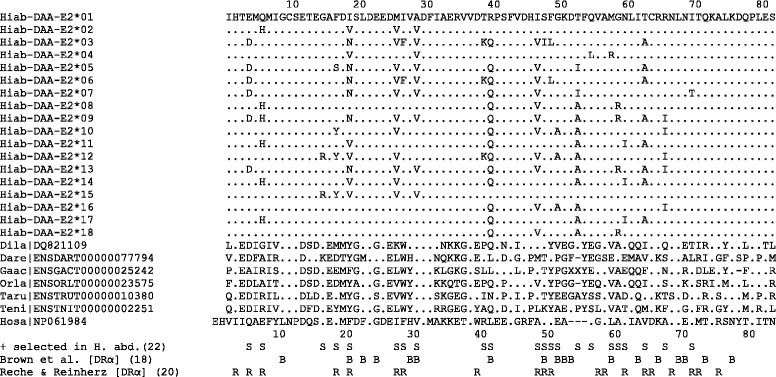
**Amino acid alignment of MH class IIα alleles.** Alignment contains all seahorse alleles (Hiab) along with a selection of teleost fish sequences (*Dicentrarchus labrax* (Dila); *Danio rerio* (Dare); *Gasterosteus aculateus* (Gaac); *Oryzias latipes* (Orla); *Takifugu rubripes* (Taru); *Tetraodon nigroviridis* (Teni)) and human (Hosa). Sites identified as being under positive selection in the seahorse, are indicated, as well as those identified as peptide-binding sites in previous crystallographic analyses of human MHC II [2,3]. Genbank/Ensembl identification codes indicated for publically available sequences. Corresponding nucleotide sequences for all seahorse MH class IIα alleles have been deposited in GenBank (KP259890-KP259907).

All 25 observed nucleotide substitutions in the MH IIα PBR were found to be non-synonymous, translating into amino acid substitutions at 22 of the 82 sites of the translated protein sequence, striking evidence of positive selection (dN/dS: ∞; dN = 0.039; dS = 0.000; Z-test for positive selection: p < 0.001; Table 2). A site-specific analysis of nucleotide variation including positive selection (M8) fit the data significantly better than a neutral model of molecular evolution (M7) (LRT = 51.507, df = 2, p < 0.001) and revealed significant evidence of positive selection at all 22 variable amino acid sites (Bayes Empirical Bayes Analysis; p > 99%; Figure 4), a pattern consistent with that observed at the physically linked MH IIβ locus, where coding variation is also concentrated at peptide-binding sites [11]. Inferences based on a comparison of the M1a (Nearly Neutral) and M2a (Positive Selection) models in Codeml were identical (data not shown).

**Table 2:** **Synonymous and non-synonymous substitution rates for exon 2 alleles of the seahorse MH class IIα gene**

**Locus**	**Length (bp)**	**Samples**	**Alleles**	**dN**	**dS**	**dN/dS**
Exon 2	246	101	18	0.039	0.000	∞**
Exon 2, PBS	60	101	17	0.110	0.000	∞*
Exon 2, non-PBS	186	101	11	0.018	0.000	∞*

Probabilities (*< 0.05, **< 0.001, ns = not significant) are derived from a Z-test (H1 = positive selection). Peptide-binding sites (PBS) refer to the human sites, identified by crystallographic analysis [3] (Figure 4).

### Network comparison reveals the existence of composite recombinants

**Figure 5:**
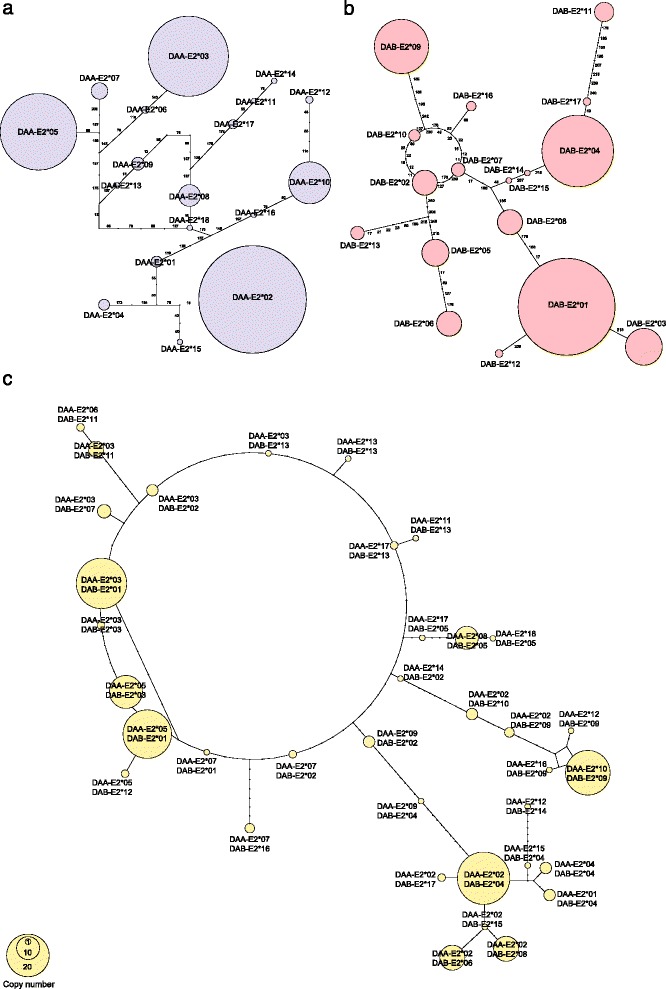
**Allelic networks of MH class II variation (n = 101 individuals).** Figure shows allelic variability in the pattern of non-synonymous substitutions across exon 2 of **(a)** MH class IIα and **(b)** MH class IIβ, the immunologically-active region of the two genes; **(c)** Composite MH network constructed from phased genotype data. Note the presence of reticulation in the MH IIβ network, evidence of intralocus recombination [11]. Reticulation is also evident in the composite allele network.

An allelic network was constructed to visualize relationships among the 18 MH IIα alleles and their relative frequencies (Figure 5a). The structure of the MH IIα network is similar to that observed at MH IIβ, with a small number of common alleles (DAA-E2*02, 03, 05 and 10), and a larger number of low frequency alleles present in ≤ 10 copies in the population sample (Figure 5).

An analysis of genetic linkage between MH IIα and IIβ alleles in the population sample supported the results of the parentage analysis, and indicated significant linkage disequilibrium between the two loci (χ^2^ = 469.70; p < 0.001). With 18 alleles detected at MH IIα and 17 alleles at MH IIβ, a minimum of 18 composite alleles and maximum of 18 * 17 = 306 alleles could be realized in the population sample. The observation of 36 composite MH IIα/IIβ alleles in the population dataset (Figure 5c) reflects a significant history of recombination in this region.

### Evidence of intra- and interlocus recombination

A statistical analysis of recombination at MH IIα failed to detect significant evidence of intralocus recombination (p = 0.077). An analysis of the phased MH IIα/IIβ dataset, in contrast, found a strong signature of recombination (p = 0.0002), with 8 MH IIα/IIβ recombinants and 2 of the 3 MH IIβ intralocus recombinant genotypes identified in an earlier analysis of MH IIβ PBR variation [11] (Table 3).

**Table 3:** **Inferred recombinant genotypes from analysis of the phased MH class IIα/IIβ PBR dataset**

**Genotype**	**Recombination breakpoint**	**Mutation savings**	**p-val**
DAA-E2*9/DAB-E2*4	198-4896	9	0.0001
DAA-E2*3/DAB-E2*13	180-4896	9	0.0002
DAA-E2*12/DAB-E2*14	188-4890	7	0.0002
DAA-E2*13/DAB-E2*13	198-4890	6	0.0001
DAA-E2*7/DAB-E2*2	210-5007	6	0.0016
DAA-E2*2/DAB-E2*6	5058-5079	6	0.0004
DAA-E2*9/DAB-E2*2	198-4896	6	0.0001
DAA-E2*2/DAB-E2*9	193-5007	5	0.0021
DAA-E2*2/DAB-E2*10	4930-5007	5	0.0001
DAA-E2*14/DAB-E2*2	180-4896	5	0.0053

Note: Recombination breakpoints for interlocus recombinants include 4630 bp separating the sequenced peptide binding regions of MHIIα and IIβ (see Figure 1).

### Protein structure

**Figure 6:**
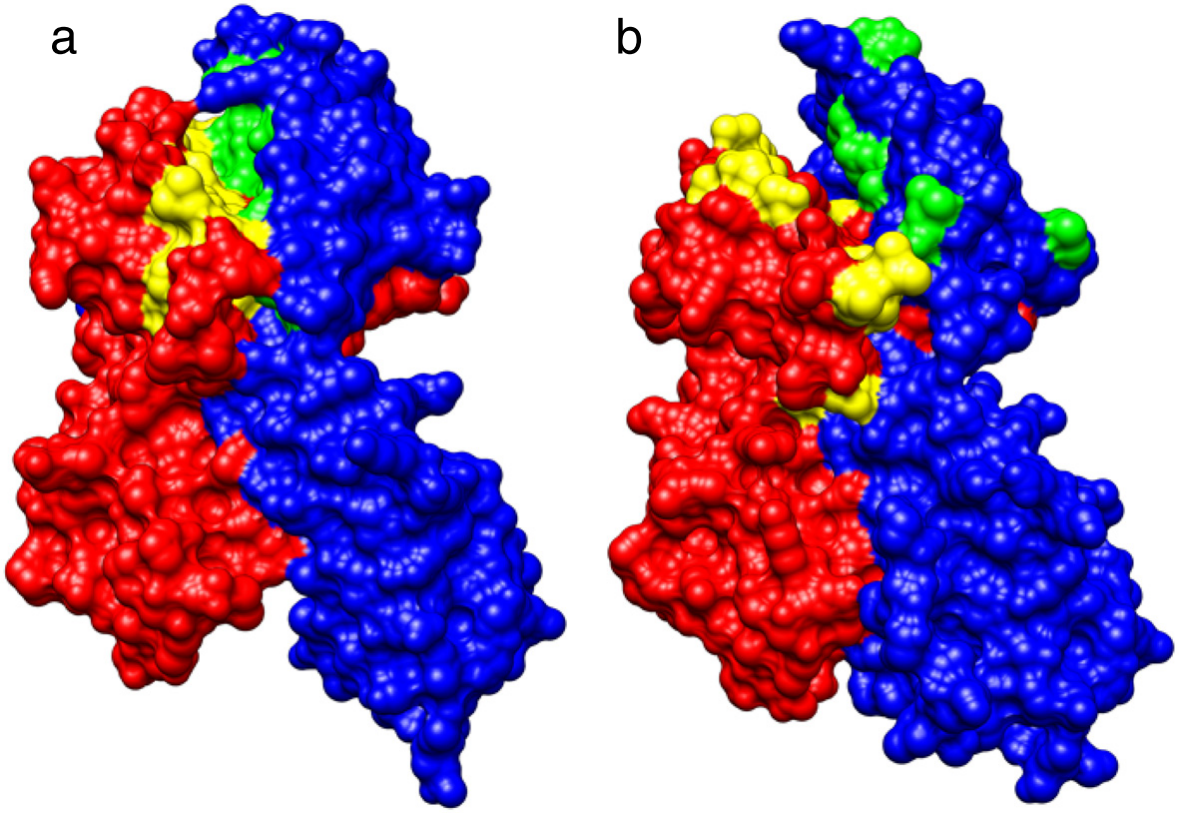
**Positively selected sites of the seahorse MH class II are concentrated in the inferred peptide binding groove of the mature protein.** Quaternary structure models of the major histocompatibility class II molecule for **(a)** *Mus musculus* and **(b)** *Hippocampus abdominalis*, based on crystallographic reconstructions for *Mus* (pdb1es0). Surface structures of extracellular components of MH IIα (red) and MH IIβ (blue) are shown, as are **(a)** peptide-binding sites, and **(b)** sites inferred to be under positive selection in the seahorse (yellow and green for MH IIα and IIβ respectively).

The inferred quaternary structure of the seahorse MH class II molecule closely resembles that of the mouse model, with a clearly defined peptide binding groove formed by the interaction of MH class IIα and IIβ loci (Figure 6). While the majority of positively selected sites in the seahorse are located in this peptide-binding groove, several selected sites at both the IIα (LYS.39α and GLN.40α) and IIβ loci (LEU.18β and ASN.61β) are located away from this groove on the surface of the mature peptide.

## Discussion

Adaptive immune diversity at the minimal MH class II system of the seahorse is enhanced by high levels of genetic variation at both MH IIα and IIβ, highlighting the importance of considering both loci in order to understand MH class II function in this system. Intra- and interlocus recombination act to further augment genotypic variation, generating novel genotypes despite the tight physical linkage of MH class II loci in the seahorse.

Despite high levels of functionally important genetic diversity and clear evidence of positive selection in the PBR of MH IIα, this region shows a complete absence of synonymous substitutions. The significant excess of non-synonymous substitutions relative to synonymous variation is also characteristic of the PBR of MH IIβ [11], and low levels of genetic variation are found in the other coding regions of the two genes [Figure 1; 12]. While the high number of non-synonymous variants in the PBR is expected given its role in antigen recognition, the low levels of synonymous substitutions suggest that selective sweeps driven by positively selected sites may act to homogenize neutral variation through a process of genetic hitchhiking [12]. New mutations at sites involved in antigen detection that provide a selective advantage in host-pathogen interactions are expected to rapidly increase in frequency [17], simultaneously increasing the frequency of linked neutral variants. Subsequent short-tract gene conversion and recombination may enhance functional diversity at MH peptide binding sites, without increasing neutral genetic variation. Over time, the hitchhiking of linked genetic variants with positively selected sites may erode local neutral variation, and impact functionally important genetic variation at neighboring loci. The combination of positive selection and genetic hitchhiking is likely responsible for the highly skewed dN/dS ratios observed here.

Amino acids experiencing positive selection at both MH IIα and IIβ are preferentially located at sites predicted to be directly involved in antigen binding (Figure 4), as inferred from homology modelling to the experimentally determined crystallographic structures of human and mouse MHC [3,18]. The presence of several positively selected sites outside this region (Figure 6) suggests potential differences in the quaternary structure of the mature protein *in vivo* and/or additional selective constraints acting away from the main peptide binding groove of the seahorse.

Despite the tight physical linkage of MH class II loci in the seahorse, both intra- and interlocus recombinants were detected in the population sample, a finding which likely reflects the selective advantages of novel allelic variants generated by recombination. While empirical recombination rates are not available for the seahorse, a rough approximation based on the average recombination rate in the human genome (ca. 1 cM/Mb) would suggest that a crossover event in the 5,000 bp interval separating the PBRs of MH IIα and IIβ would be expected every 20,000 meioses. Recombination is likely to be even more common in the MH region, as hotspots have been identified in the MH class II regions of humans [19] and mice [20] that have been shown to provide an important source of allelic variation at these loci. Sperm profiling indicates that gene conversion, the unidirectional exchange of short sequence tracts without crossover, may make up as much as 90% of all recombination events at MH class II loci [21], and serve a particularly important role in generating new PBR variants from standing genetic variation. Once new variants are produced, they may rapidly spread through the population if they offer selective advantages relative to existing allelic variation [17].

While analyses of linkage disequilibrium in population samples provide evidence of breakpoints of historical recombination events, the estimation of recombination rates requires the identification of germline recombinants from pedigree data and/or the direct typing of gametes. No MH IIα/IIβ recombinants were identified in the parentage analysis carried out as part of this study, but the modest size of this dataset had limited power to identify meiotic recombination. Although seahorses have reduced testes and produce low amounts of sperm [22], sperm typing could provide a high throughput method to estimate meiotic recombination rates, and to explore the relative importance of gene conversion and classical recombination in structuring patterns of genetic variation across the major histocompatibility region of the seahorse. An understanding of baseline recombination rates would also facilitate more detailed analyses of the potential selective advantages of novel recombinant genotypes.

Studies of MH class II genetic variability in non-model organisms have been heavily biased towards MH IIβ, an approach which has been informed by the low variability of MH IIα relative to MH IIβ in humans. Large scale population genotyping of the 5 classical MH IIα and IIβ genes in more than 20,000 individuals has identified >2,750 MH IIβ alleles and only 116 MH IIα variants, a greater than 20× excess of MH IIβ allelic diversity relative to MH IIα [6].

Gomez [9] carried out the first integrative analysis of MH IIα and IIβ variation in a non-model species, simultaneously characterizing genetic variation at both loci of the minimal MH system of salmonid fishes. An analysis of genetic variation in population samples of three salmonid species revealed similar levels of variation at both loci (MH IIα/IIβ alleles - *Oncorhynchus mykiss* (n = 40): 10/11; *Oncorhynchus kisutch* (n = 75): 4/8; *Salmo salar* (n = 27): 9/9), a result which led the authors to suggest that high genetic variability at MH IIα could allow species with a minimal MH system to mount a successful immune response without the additional genetic variation associated with multiple gene copies [9].

Recent work suggests that the high genetic variability of MH IIα may not be restricted to species with a minimal MH system such as salmonids or seahorses, but may be evolutionarily widespread. A recent population survey (n = 367 wild-caught individuals) of single copies of MH IIα (H-2A*a*) and IIβ (H-2-E*b*) in the two locus system of the house mouse (*Mus musculus*) recovered 27 MH IIα and 17 MH IIβ alleles, an excess of MH IIα allelic variability relative to MH IIβ [23]. Similarly, a large genotyping study of two MH loci in Japanese domestic cattle (n = 650) detected 15 and 26 alleles at MH IIα (DQA1) and MH IIβ (DRB3) loci, respectively [24]. Taken together, the results of these recent studies suggest that high genetic diversity at MH IIα is likely to be widespread, and is not a phenomenon restricted to species with a minimal MH class II system. As such, studies which continue to focus exclusively on variation at MH IIβ may be missing an important component of ecologically relevant variation, providing an incomplete picture of how selective pressures influence genetic diversity of the biologically active protein dimer.

Unfortunately, while it is relatively straightforward to characterize MH diversity using traditional Sanger sequencing in species carrying a minimal MH system, researchers working on more complex MH systems have been unable to accurately screen MH allelic diversity and classify variation to specific loci, an essential prerequisite for more detailed investigations of selection. Recent innovations in next-generation sequencing [25], coupled with rapidly maturing statistical approaches for processing population data for multigene families such as the MHC [26,27], offer an exciting new avenue for high-throughput systems-based analyses of immunological variation. While such studies are still in their infancy, they show great promise for population-level analyses of MH loci e.g. [28-30]. Thus far, studies have followed the design of earlier research in focusing on MH IIβ, but combined analyses of MH IIα and IIβ should be straightforward once these new technologies have fully matured.

## Conclusions

Antigen recognition by the adaptive immune system of the seahorse is enhanced by high levels of variability at both MH class IIα and IIβ loci. Strong positive selection on sites involved in pathogen recognition, coupled with high levels of intra- and interlocus recombination, generate a patchwork distribution of genetic variation essential for understanding the functional diversity of the mature protein.

While studies of MH diversity in non-model organisms are now widespread, the majority of investigations have focused exclusively on MH class IIβ, an approach that has been justified by the relatively low levels of variation observed at MH class IIα loci in humans. Our results suggest that humans may be atypical in this regard, a conclusion supported by recent comparative analyses of MH class II diversity in both mammals and fish, which indicate that genetic diversity at MH class IIα loci may often meet or exceed that observed at class IIβ. Given the high rates of intra- and interlocus recombination found at MH loci, studies aiming to link patterns of MH class II diversity to survival and reproduction should consider the structure and function of the immunologically active protein molecule. In failing to do so, researchers may be unintentionally overlooking an important component of ecologically relevant genetic variation.

## Methods

### Sample collection

Adult seahorses (*H. abdominalis*) were purchased from a commercial breeding facility (Seahorse Australia, Beauty Point, Tasmania) and held at the University of Zurich under an animal care and experimentation permit from the Veterinäramt Zürich (Permit 103/2008). In addition, fin clips from 5 wild-caught seahorses from Sydney, Australia (2 individuals collected in 2003) and Tasmania (3 individuals collected from 3 populations in 2003 and 2004) [31] were included in analyses of MH class II genetic diversity.

### Gene discovery via high-throughput transcriptome profiling

A full plate of 454 sequencing of transcriptome libraries prepared from pouch and reference (brain, gills, liver, heart, kidney and testes) tissues from a single pregnant and non-pregnant seahorse using the GS FLX Titanium Chemistry (Roche) recovered six partial transcripts of the MH IIα locus of the seahorse, which were assembled into a single 541 bp contig spanning the 5′ UTR, complete exons 1 and 2 and partial exon 3. Further details on this transcriptome screen are available elsewhere [11]. A TBLASTX search of the nucleotide collection of GenBank identified MH class IIα of *Dicentrarchus labrax* as the top hit (DQ821109.1: e-value = 3e-47), followed by class IIα genes from other teleost species (*Morone saxatilis* L35062.1: e-value = 8e-42, *Larimichthys crocea* EF681861.1: e-value = 5e-41, *Miichthys miiuy* GU936787.1: e-value = 2e-40, and *Epinephelus coioides* GU992883.1: e-value = 3e-40).

### Full-length cDNA sequencing

Total RNA was extracted from brain, gill, kidney, liver, pouch and testes tissues of two pregnant seahorses with RNeasy extraction columns (Qiagen). RNA extractions were subsequently DNase treated, standardized to a common concentration of 85 ng, and pooled for library construction. 5′ and 3′ RACE libraries were prepared from 1 μg of total RNA using a SMARTer RACE cDNA amplification kit (Clontech).

The full-length cDNA sequence of the MH class IIα gene of the seahorse was obtained using separate 5′ and 3′ RACE reactions primed with gene-specific primers (MHIIαE2F and MHIIαE3R; Table 1), in 25 μL PCR reactions using 1.5 μL of 5′/3′ RACE-ready cDNA. Both reactions produced single products from the 5′ and 3′ ends of the class IIα gene, which were PCR-purified with Montage PCRμ_96_ Filter Plates (Millipore) and eluted in 20 μL ddH_2_0 in preparation for sequencing.

Sequencing reactions were carried out in 10 μL volumes consisting of 2–4 μL purified PCR product, 0.2 μM primer, and 1 μL Big Dye v3.1 Terminator Cycle Sequencing mixture (Applied Biosystems). Cycling conditions consisted of 30 cycles of 10 sec at 96°C, 5 sec at 50°C and 4 min at 60°C. Ethanol-purified products were sequenced on an ABI 3730 automated sequencer (Applied Biosystems).

### Full-length genomic sequencing of the Major Histocompatibility class II gene region

To elucidate the structure and distribution of variation across the MH region of the seahorse, genomic DNA of a single non-pregnant individual was extracted from muscle tissue (DNeasy, Qiagen). The quality of extracted DNA was assessed on a 1.5% agarose gel and spectrophotometrically quantified using a Nanodrop 2000 (Thermo Scientific). The gDNA sequence of the MH class IIβ for this individual has been previously published [12].

The full-length gDNA sequence of the seahorse MH class II gene region was determined using long-range PCR with gene-specific primers designed from the cDNA sequence of MH class IIα and the 5′ UTR region of the class IIβ locus (MHIIα-E1F3/MHIIβ-5UTRR4; Table 1).

Long range PCR was performed in a 25 μL volume containing 3U LongAmp Taq (New England Biolabs), 1× LongAmp reaction buffer, 0.4 mM dNTPs, 0.2 μM primers and 250 ng DNA. PCR amplification involved a 2 min denaturation step at 92°C, followed by 30 cycles of 92°C (20 s), 65°C (20 s) and 65°C (10 min), and a final extension step for 10 min at 65°C. The PCR reaction was filter-purified in preparation for cloning.

4 μL of purified PCR product was cloned into a TOPO TA cloning vector (Invitrogen) following the manufacturers′ recommendations. Following overnight culture of transformed chemically competent *E. coli* at 37°C, 5 positive colonies were picked and grown for 16 h in liquid culture on a 200 rpm horizontal shaker at 37°C. Liquid cultures were purified for downstream sequencing using a QIAprep Spin Miniprep kit (Qiagen).

Screening of plasmid DNA with primers for the hypervariable peptide binding region of the seahorse MH class IIα peptide binding region revealed the presence of two alleles, both of which were sequenced to completion using a nested sequencing strategy involving primers distributed across the full length of the amplified region (Table 1) using the protocols outlined above. DNA sequencing revealed a 786 bp deletion between MH class IIα and IIβ in one of the two full-length alleles (Figure 1).

The intervening non-coding sequence between the class IIα and IIβ loci was PCR-amplified and sequenced from genomic DNA using two sets of PCR reactions, one using MHIIα-E4F/MHIIβ-5UTRR2 (60°C anneal) and a second using MHIIβ-5UTRF2/MHIIβ-E2R2 (55°C anneal), both of which spanned the deletion region, allowing the determination of allelic phase of sequences from IIα and IIβ loci.

### RT-PCR screening of tissue-specific expression

Samples were obtained from captive *H. abdominalis* individuals (Seahorse Australia, Beauty Point, Australia) preserved in RNAlater (Sigma-Aldrich) and then stored at −80°C. Four reproductively active adult males were screened for MH II gene activity, with the stage of pregnancy estimated using a recently published developmental key for syngnathid fishes [32]. Total RNA was extracted from a panel of tissues (brain, gill, heart, kidney, liver, pouch, testis from one mid-pregnant animal; and pouch from one non-pregnant, one early pregnant, and one late pregnant individual) using an RNeasy Mini Kit with QiaShredder (Qiagen) and DNase I (Invitrogen) digestion. First-strand cDNA was synthesized with SuperScript III First-Strand Synthesis System for RT-PCR (Invitrogen) using random hexamer priming and 200 ng of RNA.

MH class II PCRs were carried out using intron-spanning primers indicated in Table 1. Beta-actin (ACTB) was used as the positive control to ensure uniform amplification for each tissue, and was amplified using ACTB-E2F - GTCATGGTCGGCATGGGAC and ACTB-E3R - AGGTAGTCTGTGAGGTCTCG. PCR reactions for ACTB were performed in 20 μl volumes containing 0.5U Taq (New England Biolabs), 1× NEB reaction buffer, 0.75 μM MgCl_2_, 0.2 mM dNTPs, 0.2 μM primers, and 1.0 μl DNA, with the following PCR cycling conditions: 95°C for 1:30, then 30 cycles of 95°C for 0:30, 53°C for 0:30, 68°C for 1:00, followed by 68°C for 5:00. MH amplifications were performed in 25 μl volumes containing 1U Taq (NEB), 1× NEB reaction buffer, 1.0 μM MgCl_2_, 0.4 mM dNTPs, 0.2 μM primers and 2.5 μl DNA, with the following cycling parameters: 92°C for 0:10, then 40 cycles of 92°C for 0:10, 55°C for 0:30, 68°C for 2:00 (for MH IIα) or 92°C for 5:00, then 40 cycles of 92°C for 0:30, 62°C for 0:30, 68°C for 4:00, followed by 68°C for 15:00 (for MH IIβ).

PCR products were subjected to electrophoresis at 100 V for 20 min in 1.5% agarose gels stained with ethidium bromide, and visualized using an AlphaImager gel documentation system (Alpha Innotech).

### MH IIα inheritance and MH II linkage analysis

MH class IIα Exon 2, containing the immunologically active peptide-binding region of the gene, was PCR-amplified and sequenced in a sample of 47 F1 individuals from 5 families (n = 8–13 per family) which had previously been characterized for patterns of genetic variation at the MHIIβ peptide-binding region [11]. A comparison of parent-offspring genotype profiles allowed the inference of the mode of MHIIα inheritance and a means to test for linkage of IIα and IIβ loci in this species.

Standard PCR was performed in 25 μL volumes containing 1U Taq (NEB), 1× NEB reaction buffer, 1.0 μM MgCl_2_, 0.4 mM dNTPs, 0.2 μM of either MHIIα-E2F/MHIIα-E3R or MHIIα-I1F3/MHIIα-E3R and 25–250 ng DNA. PCR amplifications involved a 10 s denaturation step at 92°C, followed by 40 cycles of 92°C (10 s), 55°C (30 s) and 68°C (2 min). All individuals were PCR-amplified and sequenced using both sets of primer pairs. PCR amplifications of both PCR purification and sequencing followed that outlined for the cDNA experiment above, producing the full length sequence of the 249 bp exon. After trimming 2 bp from the 5′ end of the sequence alignment and 1 bp from the 3′ terminus to exclude incomplete amino acids, the analyzed exon 2 dataset included 246 bp/82 amino acids.

### Characterization of the MH IIα peptide-binding region (PBR)

Exon 2 of MH class IIα was also sequenced in a population of 101 seahorse individuals for which the peptide-binding region of the MH class IIβ locus had previously been characterized [11] to obtain an estimate of population-level variability of this region. PCR amplification and sequencing conditions were identical to those outlined above.

### Sequence processing

All PCR reactions were sequenced in both directions, aligned using ClustalW [33] and visualized in BioEdit v.7.0.9 [34]. Heterozygous sites were coded using IUPAC nomenclature for degenerate positions, and allelic sequences were inferred using the default settings of PHASE V2.1.1 [35]. Individuals for which allelic phase could not be reliably inferred by statistical inference (Phase probabilities ≥ 0.95) were re-amplified and cloned (MH IIα: 1 individual, MH IIβ: 1 individual, MH IIα/MH IIβ: 4 individuals). Four to five colonies were sequenced from each cloned individual, allowing the direct determination of individual alleles. All private alleles were separately re-amplified and sequenced to verify their identity (MH IIα: 6 individuals, MH IIβ: 2 individuals, MH IIα/MH IIβ: 13 individuals).

### Analyses of sequence polymorphism/linkage

Nucleotide diversity (π) at MH IIα was estimated under the maximum composite likelihood model implemented in Mega v6.0 [36], with standard error estimates derived from 500 bootstrap replicates. Exact tests of Hardy-Weinberg equilibrium (1,000,000 step Markov Chain, 100,000 dememorization steps) were performed in Arlequin v3.5.1.2 [37].

Analysis of gametic phase of MH IIα and IIβ genotypes was performed using the Bayesian ELB approach [38] implemented in Arlequin v.3.5.12. Pairwise linkage analysis of unphased MH IIα and IIβ data was also carried out using Arlequin (20,000 permutations, 5 EM replicates).

### Site-specific tests of positive selection

Characterization of synonymous and non-synonymous substitutions across the peptide binding region of MH IIα was performed in Mega v6.0 [36] using the Nei-Gojobori method with Jukes-Cantor distances. A one-tailed Z-test of positive selection (500 bootstrap replicates) tested the null hypothesis of neutral evolution for putative peptide binding sites, non-binding sites, and the full peptide binding region.

A neighbor-joining tree was constructed from MH IIα alleles using the maximum composite likelihood method implemented in Mega v6.0 [36]. This tree served as a starting tree for a site-specific analysis of positive selection in Codeml v4.8 [39], which compared the fit of a neutral evolution model with recombination (M7) with one allowing for positive selection (M8), using a likelihood-ratio test (LRT). Sites experiencing positive selection were identified following a Bayes Empirical Bayes analysis (posterior probability ≥ 0.95) [40].

### Network construction

An allelic network was constructed to visualize genetic relationships among alleles of the MH IIα PBR using TCS v.1.21 [41], and prepared for publication using yED v3.12.2 [42]. A second network was constructed using phased MH IIα and IIβ data, in order to visualize the frequency and distribution of MH class II composite genotypes.

### Recombination

The presence of recombination in the seahorse MH class II region was investigated using RECCO v.0.93 [43] (10,000 permutations), using a minimum mutation savings criterion of 5 to identify recombinants. Recombination analyses were carried out independently for the MH IIα PBR dataset, and for a concatenated alignment of phased MH IIα/IIβ data, allowing the identification of intra- and interlocus recombination. Inferred recombination breakpoints for interlocus recombinants included an offset of 4,630 bp of unknown sequence separating the PBRs of the two genes (Figure 1), reflecting uncertainty in the location of breakpoints in the unsequenced region between the two PBRs.

### Protein structure

The quaternary structure of the MH class II complex of the seahorse was reconstructed via homology modeling of the full-length MH class IIα and IIβ loci to the previously determined crystallographic structure of the mouse MH class II molecule, using Protinfo PPC [44]. Inferred protein surface models of target and database sequences were annotated and visualized in Chimera v1.6.2 [45].

Protinfo PPC returned five significant hits (structure confidence: 41-55%), all of which matched PDB models for the extracellular domain of the MH class II complex of *Mus musculus*. One of the three top hits (PDB ID: 1ES0: structure confidence 55%) was selected as a model for the seahorse MH class II complex. The expression vector, peptide and linker of the mouse structure were omitted from the modeled data, as well as 8 aa of MH IIβ not resolved in the original model, resulting in a total of 182 aa and 180 aa for the MH class IIα and IIβ loci, respectively. Known peptide-binding sites for the human MH class II molecule [3] were annotated on the mouse model, along with sites under positive selection in the seahorse.

### Availability of supporting data section

Sequence data generated for this project have been deposited in GenBank (Accession #: KP259890-KP259909).
